# Neuroprotection in Stroke—Focus on the Renin-Angiotensin System: A Systematic Review

**DOI:** 10.3390/ijms23073876

**Published:** 2022-03-31

**Authors:** Sebastian Andone, Zoltan Bajko, Anca Motataianu, Smaranda Maier, Laura Barcutean, Rodica Balasa

**Affiliations:** 1 Doctoral School, ‘George Emil Palade’ University of Medicine, Pharmacy, Science, and Technology of Târgu Mures, 540142 Târgu Mures, Romania; sebastian.andone@umfst.ro (S.A.); rodica.balasa@umfst.ro (R.B.); 2 Ist Neurology Clinic, Mures County Clinical Emergency Hospital, 540136 Târgu Mures, Romania; anca.motataianu@umfst.ro (A.M.); smaranda.maier@umfst.ro (S.M.); laura.barcutean@umfst.ro (L.B.); 3 Department of Neurology, University of Medicine, Pharmacy, Science and Technology Târgu Mures, 540136 Târgu Mures, Romania

**Keywords:** stroke, neuroprotection, renin-angiotensin

## Abstract

Stroke is the primary cause of disability in the adult population. Hypertension represents the leading risk factor being present in almost half the patients. The renin-angiotensin system is involved in the physiopathology of stroke and has an essential impact on hypertension as a risk factor. This article targeted the role of the renin-angiotensin system in stroke neuroprotection by reviewing the current literature available. The mechanism of action of the renin-angiotensin system was observed through the effects on AT1, AT2, and Mas receptors. The neuroprotective properties ascertained by angiotensin in stroke seem to be independent of the blood pressure reduction mechanism, and include neuroregeneration, angiogenesis, and increased neuronal resistance to hypoxia. The future relationship of stroke and the renin-angiotensin system is full of possibilities, as new agonist molecules emerge as potential candidates to restrict the impairment caused by stroke.

## 1. Introduction

Stroke is the leading cause of long-term disability in developed countries and one of the top causes of worldwide mortality [1].

According to the latest available statistical data regarding the incidence of stroke in the US, approximately 795,000 patients present with stroke every year, of which approximately 25% are recurrent strokes. From the total number of reported strokes, 87% were reported ischemic [2].

More than 90% of the risk of developing stroke can be attributed to modifiable risk factors such as arterial hypertension, hyperglycemia, hyperlipidemia, or behavioral risk factors such as smoking or sedentarism [3].

From these risk factors, arterial hypertension represents the most important one due to its presence in approximately 50% of patients who develop stroke [4,5].

Not only that, but hypertension represents a significant risk factor for other cerebral pathologies that produce cognitive impairment, including Alzheimer’s disease. Increased blood pressure values have consequential effects on cerebral circulation, especially cerebral microcirculation. Because of this, hypertrophic remodelating of the arterial wall appears, which causes an increased media thickness and a reduction of the lumen diameter [6].

Apart from the structural changes, hypertension also increases collagen production, leading to increased arterial wall rigidity, a predictive factor for both stroke and cognitive impairment [7].

In the large vessels, hypertension promotes atherosclerosis, creating another stroke risk factor. This can evolve to an acute ischemic stroke either by plaque rupture and acute local thrombosis or artery-to-artery embolism [8].

In addition to other frequent causes of stroke, such as cardioembolic, large vessel disease, or small vessel disease, hematological disorders can also be attributed as uncommon etiology [9].

Blood pressure control improved in the last decades, decreasing the incidence of stroke in this specific group of patients, although hypertension prevalence is rising.

This was possible primarily because of improved therapeutic management and the development of new drugs with more clinical potential [10].

The role of antihypertensive treatment is well established by reducing the incidence of stroke. The renin-angiotensin system (RAS) appears to be involved in acute ischemic stroke etiology and is connected to other risk factors involved in stroke pathogenesis. Therefore, it is remarked as a potential therapeutic target for preventing stroke [11].

The RAS has two places of action with an effect during stroke: cerebral parenchyma, where the anti-inflammatory and neuroprotective properties are exploited, and cerebral vascularization, affecting blood flow [12].

The actions of RAS are mediated by two main types of receptors: angiotensin II type 1 (AT1) and angiotensin II type 2 (AT2).

Both AT1 and AT2 receptors belong to the protein G superfamily, but differ as expression and signaling pathways.

AT2 receptors appear mainly in the fetal period and their number decreases quickly after birth, but they can be identified in the vascular endothelial tissue, myocardial tissue, and central or peripheral nervous system. By contrast, AT1 receptors appear mostly in the adult organism [13].

Angiotensin I, also called pro-angiotensin, is formed by the action of renin over angiotensinogen, an alfa-2-globulin, synthesized in the liver. Angiotensin I appears to have no biological action, playing only the role of a precursor for angiotensin II, the conversion taking place in the lungs through the action of the angiotensin-converting enzyme. The importance of the angiotensin-converting enzyme has recently hit the spotlight, primarily due to its role in the pandemic with novel coronavirus SARS-CoV-2 in the physiopathology of multiorgan failure associated with the infection [14].

## 2. Methods

We undertook a systematic review following PRISMA (Preferred Reporting Items for Systematic Reviews and Meta-analyses) guidelines (http://www.prisma-statement.org/ (accessed on 1 December 2021)).

The search strategy included two journal databases: NCBI PubMed and Google Scholar.

We performed a query using the search terms: stroke + neuroprotection + (renin OR angiotensin OR renin-angiotensin).

**Inclusion criteria**: we included studies that provided statistical analysis (descriptive statistics, comparison tests, categorical data tests, linear regression, uni- and multivariate statistics), original articles, and reviews regarding stroke and angiotensin.

**Exclusion criteria**: we excluded articles that focused only on the systematic effects of angiotensin without any mention of central nervous system effects.

**Figure 1:**
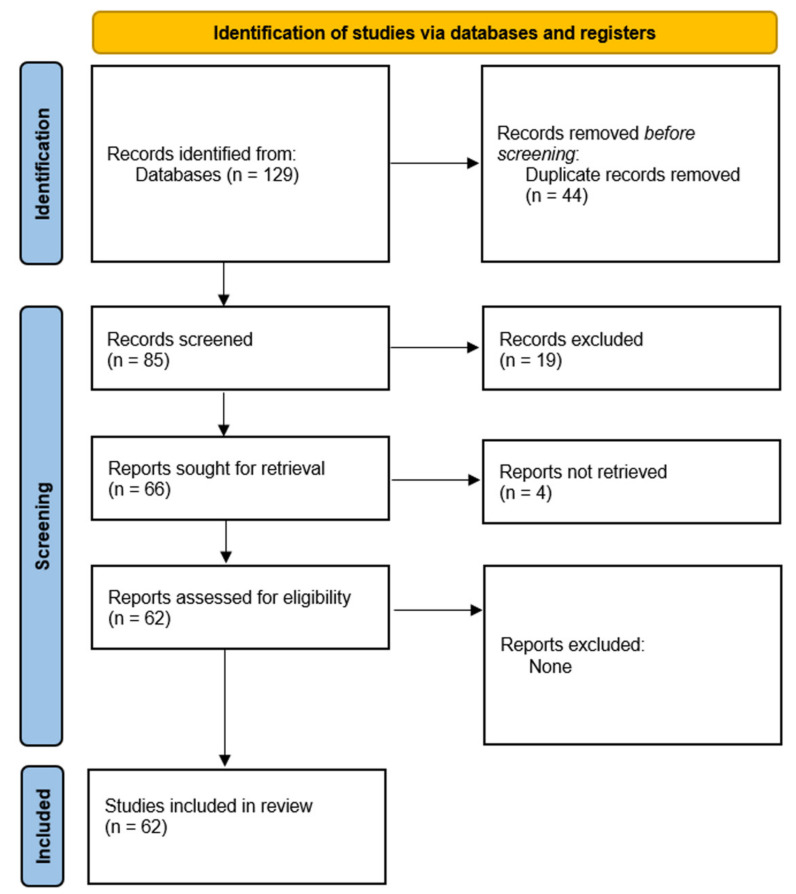
PRISMA flow diagram for the systematic review.

After screening, duplicate removals, and exclusions, we identified a total of 127 studies that we included into our review (Figure 1).

## 3. Results and Discussion

### 3.1. Angiotensin Receptors: AT1, AT2 and Mas

Angiotensin (ANG) II is involved in the physiopathology of stroke and regulates hydric balance, sodium uptake, thirst, arterial blood pressure, and cognitive functions at the brain level, affecting cerebral vascularization self-regulation and inhibiting endothelial-dependent relaxation [15,16].

The most significant clinical contribution is to arterial hypertension, a risk factor in cerebrovascular diseases that affects the local vascular regulatory mechanisms, vascular growth, and permeability [17].

Angiotensin II receptors are located at the level of the nuclei and areas corresponding to the autonomic nervous system [15,16]. Angiotensin II increases immediately after a stroke in the cortex and hypothalamus [18].

AT2 receptors are upregulated at the level of tissular injury, both in the brain parenchyma and myocardial tissue. This upregulation is vital for the sustenance of cerebral circulation and has a role in cellular repair and regeneration. AT2 receptor stimulation also allows cerebral vasodilation and has an anti-inflammatory effect [19,20].

Another receptor with a neuroprotective role is the AT4 receptor. This receptor is unlike its sibling receptors AT1 and AT2 from a structural point of view. It is linked to an insulin-regulated aminopeptidase near the glucose transporter GLUT4.

Not only do AT4 receptors have a role in cerebral vasodilatation, but they also promote the increased uptake of glucose [21].

Inside the cerebral parenchyma, activation of the ACE2/Ang-(1-7)/Mas system could be responsible for the anti-inflammatory and neuroprotective action, while Ang-(1-7) can block the inflammatory reaction of the glia.

**Figure 2:**
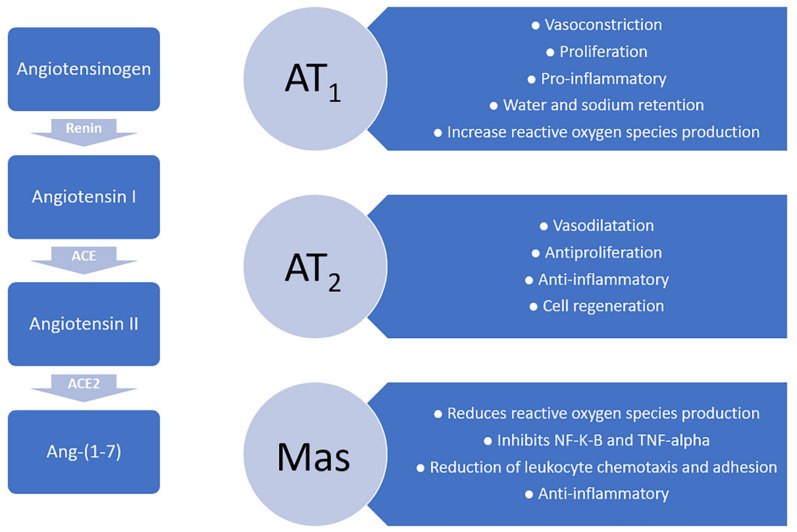
Angiotensin receptors and their roles.

The AT2 receptor has connections to other RAS and Mas receptor mediators activated by Ang-(1-7) [11] Figure 2.

### 3.2. Angiotensin-Induced Neuroprotection Effects

Angiotensin II influences cerebral circulation through its receptors, AT1, AT2, and AT4. The main effects of angiotensin II on AT1 receptors are cerebral vasoconstriction, increased secretion of reactive oxygen species, and pro-inflammatory effect.

All these lead to raised blood pressure and the disruption of the cerebral circulation, which will enable cerebral ischemia and cellular apoptosis. AT1 receptor activation will influence the infarction volume growth and involve the penumbra’s injury [22,23,24].

Angiotensin II receptor blockers have shown multiple neuroprotective effects, both through their central level action and their peripheric level action.

Besides the neuroprotective effects from stroke, other neuroprotective effects were discovered in the context of neurodegenerative diseases, such as Alzheimer’s disease, where control over risk factors like hypertension can delay the condition progression, maintain cognitive function, and reduce neurotoxic inflammation [25,26].

Another neurodegenerative disease, Parkinson’s disease, also seems to benefit from the neuroprotective effect of angiotensin II receptor blockers, primarily by protecting dopaminergic cells against injury [27].

Neuroprotective effects have been described in other pathologies like depression, anxiety, and mood changes. Another advantage of the central action of angiotensin II receptor blockers is the ability to act without passing the blood-brain barrier.

Direct control over cerebral circulation is achieved by blocking AT1 receptors in the cerebrovascular endothelium, both in large arteries and cerebral microcirculation.

Activation of AT2 receptors post-stroke stimulates neuronal growth and increases neuronal survival under hypoxic conditions.

The blockage of AT1 receptors has a proven role in neuronal protection during hypoxia through a decrease in oxidative stress [28].

The mechanism of brain hypoxia tolerance was also hypothesized as a neuroprotective effect found in patients that developed a transient ischemic attack before suffering a non-lacunar ischemic stroke. The effect was mainly suggested by the early good prognosis of these specific patients [29].

Increase of angiotensin level during the inhibition period of AT1 receptors will affect the levels of angiotensin I degradation and angiotensin II which produces biological function through different subtypes of receptors. These peptides contain fragments of angiotensin 1-7, 2-8 (angiotensin 3), and 3-8 (angiotensin 4), which act upon new receptors such as AT (1-7) and AT4 [4,30].

Ang-(1-7) activation decreases oxidative stress and limits neuronal death by reducing the secretion of nitric oxide [31].

The activation of Mas decreases oxidative stress and inhibits the nuclear Kappa-Light-Chain-Enhancer factor of activated B-cell lymphocytes (NF-K-B) and TNF-Alpha [32]. The inhibition of NF-K-B by Ang-(1-7) was proven to prevent the upregulation of vascular adhesion protein-1 by angiotensin II as well as the reduction in leukocyte chemotaxis and adhesion [33].

The stimulation of production and activation of angiotensin-converting enzyme by administration of diminazene aceturate before or after a stroke showed potential beneficial neuroprotective effects, but without altering blood pressure. The effect was utterly reversed afterward by administering a blocker agent of Mas receptors [34].

Another neuroprotective mechanism of angiotensin II receptor blockers is the integrity maintenance of the blood-brain barrier by safeguarding against the effects of hypertension and diabetes mellitus, preventing the infiltration of macrophages and the passage of injury-circulating agents over the cerebral parenchyma [35,36].

In studies performed on genetically modified mice, the ischemic lesion was reduced in mice without AT1 receptors and increased in size in mice with AT2 receptors gene deletion. Additionally, in mice with AT2 genetic defects, an increase in ischemic deterioration was observed [37,38].

Activation of RAS plays an essential role in vascular inflammation, generating reactive oxygen species and altering endothelial function.

Angiotensin II is capable of initiation and maintenance of several mechanisms which contribute to endothelial dysfunctionality, leading to a rise in cardiovascular risk. 

Atherosclerosis is linked with the production of pro-inflammatory cytokines such as IL-1, TNF-Beta, and IL-6, which are upregulated by the activation of AT1 receptors.

Angiotensin II can induce the activation of MMP, with a critical role in the rupture of atherosclerotic plaques either through direct action or by stimulation of IL6.

Angiotensin II maintains the inflammatory pro-atherogenic status through the increased expression of adhesion molecules expression. In addition, it increases the production of oxygen free radicals in the vascular wall, promotes LDL-cholesterol oxidation, and increases its uptake in the endothelial cells [15].

Activation of RAS (renin-angiotensin system) not only induces vasoconstriction and the formation of MMP and extracellular matrix, but also stimulates the migration and the proliferation of smooth muscle vascular cells, increases PAI-1 synthesis, and stimulates the pro-inflammatory cytokine release [39].

RAS is involved in vascular remodeling, left ventricular hypertrophy, and generating oxidative stress, and has a role in inflammation in the atherosclerotic process.

Angiotensin receptor blockers reduce the frequency of atrial fibrillation and stroke and prevent cardiovascular and renal events in diabetic patients [40].

Genetic polymorphism represents a modern subject of study which has not been fully explored in the domain of the renin-angiotensin system.

Even so, in a study performed on ischemic stroke patients where they studied the genotypes of the angiotensin-converting enzyme gene, a specific allele was observed to be more frequently associated with lacunar stroke, concluding that it is a risk factor for this specific stroke subtype. The same genotype was not associated with an increased risk for other stroke subtypes, such as large vessel disease [41].

### 3.3. Antihypertensive Drugs in Stroke: Focus on Angiotensin-Converting Enzyme Receptors and Angiotensin II Receptor Blockers

ACE inhibitors produce different pharmaco-dynamic effects over AT2 receptors than angiotensin receptor blockers.

ACE inhibitors reduce the generation of angiotensin II and stimulate AT1 and AT2 receptors.

The inhibition of AT1 receptors increases the production of angiotensin II and stimulates AT2 receptors by inhibiting negative feedback and releasing renin [4].

Both angiotensin-converting enzyme receptors (ACE), as well as angiotensin II receptor blockers, showed therapeutic potential in stroke.

When comparing different antihypertensive drug classes, ACE inhibitor or calcium-channel blocker treatment offers superior protection of the risk of stroke than diuretic/beta-blockers treatment or placebo.

Angiotensin II receptor blockers have a double action by blocking AT1 receptors and facilitating the stimulation of AT2 and AT4 receptors by angiotensin II. By doing so, the protective effect offered by this drug class against strokes seems to be independent of its antihypertensive effect. Multiple meta-analysis studies also suggest the same hypothesis. Furthermore, in the same studies, it is shown that angiotensin II receptor blockers have a superior neuroprotective effect to other drug classes that decrease the production of angiotensin II, such as ACE inhibitors and beta-blockers [42].

However, a couple of clinical trials could not prove this superior effect of angiotensin II receptor blockers over ACE inhibitors.

The superiority of the neuroprotective effect of angiotensin II receptor blockers over ECA inhibitors was shown in experimental studies performed on rats with middle cerebral artery occlusion, which received either Candesartan or Ramipril before the event. The infarct volume was reduced in the group treated with Candesartan but not in the group treated with ACE inhibitor [43].

Even if we compare ACE inhibitors and angiotensin II receptor blockers with other antihypertensive drugs, such as diuretics, beta-blockers, or even placebo, they have a stroke prevention effect. The same cannot be stated when comparing them with calcium channel blockers. Although ACE inhibitors are superior to calcium channel blockers in preventing coronary heart disease, it appears that ACE inhibitors are inferior when compared to stroke prevention [44].

No additional benefit was observed when comparing angiotensin II receptor blockers to calcium channel blockers. Quite the opposite, in fact: some studies have reported that blood pressure in patients treated with angiotensin II receptor blockers is higher than patients treated with calcium channel blockers, so we could conclude that the risk of developing a stroke is higher in the group treated with angiotensin II receptor blockers [45].

Several clinical studies followed the effects of routine hypertension treatment with angiotensin II receptor blockers and ACE inhibitors and compared these with other antihypertensive drug classes.

Among these, the LIFE trial compared Losartan against Atenolol treatment, and observed a reduction of stroke incidence by almost a quarter in the group treated with Losartan [46].

Another trial, MOSES, compared another angiotensin II receptor blocker, Eprosartan, with a calcium channel blocker, Nitrendipine. During a follow-up of two and a half years, the number of cardiovascular events and the number of patients that developed strokes was reduced in the Eprosartan group. It is worth mentioning that there were no significant differences in the potency of blood pressure reduction between the two drugs [47].

Using ACE inhibitors was associated with reducing stroke incidence compared to placebo.

In an unrandomized clinical trial, the relationship between ACE inhibitor treatment and stroke severity was studied, as measured by the NIH stroke scale. It was observed that patients that received treatment with ACE inhibitors prior to the stroke had a less-severe stroke when compared to patients that did not receive the treatment. Additionally, on the MRI-DWI sequence, cerebral infarcts were smaller in the group treated with ACE inhibitors [48].

In another study, stroke severity was followed in patients who received ACE inhibitors prior to the event, but this time along with antiplatelet and statin treatment, obtaining similar results. The naive patients had increased stroke severity and an increased infarct volume compared with the patients who received prior treatment [49].

ACE inhibitors aid cerebrovascular self-regulation, which is believed to have a central role in the neuroprotective effect against strokes [50].

The neuroprotective effect of angiotensin receptor blockers does not have a clearly stated mechanism—it could be connected to a reduction in the activation of AT1 receptors or the unposed activation of AT2 receptors. Evidence showed that the activation of AT2 receptors has a beneficial action in strokes, and that the angiotensin effect over this receptor has the opposite effect on the AT1 receptors.

There is evidence that supports the idea that the activation/stimulation of AT2 receptors drives neuronal differentiation and regeneration and has a neurotrophic role, which leads to the theory that the upregulation of the AT2 receptor expression in the peri-infarct cerebral areas can be activated by the endogenous increase of angiotensin II by treatment with angiotensin-receptor blockers, which could be associated with the neuroprotective effect [51].

Angiotensin-receptor blockers attenuate vascular inflammation and increase endothelial vasodilatation capacity by releasing nitric oxide and prostacyclin. Also, the angiotensin-receptor blocker treatment can improve vascular reactivity in the cortex through functional and structural alterations [13].

The protection given by angiotensin receptor blockers can be extended from the acute period towards the post-ischemic period. Irbesartan improved the neurological outcome in rats, even when it was administered after the cerebral ischemia [44].

### 3.4. Clinical and Experimental Studies from the Prism of Neuroprotection

Although many experimental studies have shown the neuroprotective potential of angiotensin II receptor blockers, its clinical potential could not be demonstrated. On the contrary, studies show a harmful effect on ischemic stroke patients [52,53].

Other pharmaceuticals have also shown neuroprotective potential, such as citicoline, which failed to prove its efficacy in human trials in stroke patients with moderate-to-severe deficits [54].

After the stroke, AT2 receptors at a neuronal level have been uniformly upregulated in the surrounding area of the cerebral infarction, and were responsible for the sparing action of the infarction given by Irbesartan [28].

Infusion of angiotensin 2 in gerbils has reduced post-experimental stroke mortality by enhancing cerebral perfusion recuperation [55].

A similar protective effect has been obtained with Losartan treatment, whereas Enalaprilat has blocked the protective action given by the AT1 receptor inhibition [56].

Angiotensin II administration at the level of cerebral arterioles in rabbits has led to endothelial-dependent vasodilatation, which Losartan diminished [57].

Administration of Ang-(1-7) in mice with ischemic stroke led to the attenuation of microglial activation markers and neuronal-derived chemokines at the cortex level [11].

After the temporary occlusion of middle cerebral artery (MCA), treatment with Ang-(1-7) not only reduced nitric oxide secretion but also decreased the level of C-X-C motif ligand 12 (CXCL12) chemokine at 6 h after occlusion, and the level of IL1-beta, IL6, and cluster differentiation 11B (CD11B) at 24 h [58].

In another study in which oral Ang-(1-7) was administered in mice after MCA occlusion induced by endothelin-1, a reduction by 25% of cerebral infarction volume was noted, with an improvement of neurological function, but with no effect on arterial blood pressure, heart rate, or cerebral blood flow [59].

Central administration of Ang-(1-7) in hypertensive rats with hemorrhagic stroke increases life expectancy and improves neurological deficit, decreasing the number of microglia by decreasing the cerebral inflammation [60].

In another study trying to find a link between the RAS and microglial activation, it was proven that in rats that received Ang-(1-7), a reduction of microglial activation could be observed. This effect was probably possible through the direct action of Ang-(1-7) on Mas receptors present on the microglia. The stimulated Mas receptors from the microglia could potentially inhibit the activation and reduce the production of pro-inflammatory cytokines. 

During a stroke, central administration of Ang-(1-7) has diminished the raised pro-inflammatory markers at the cortical level [58]. 

Ang-(1-7) has also raised bradykinin levels at the level of the cerebral parenchyma after stroke. Intracerebroventricular administration of ANG-(1-7) did not alter systolic arterial pressure.

Intracerebroventricular administration of Ang-(1-7) in rats for seven days previous to MCA occlusion induced by ET1, and followed up for three days after a stroke, led to a significantly lower cerebral infarction size. The reduction in the size of cerebral infarction induced by Ang-(1-7) was inversed by the co-infusion with Mas A779 antagonist [34].

Administration of i.v. bolus treatment with Candesartan 2 h before MCA occlusion in hypertensive rats increases cerebral blood flow in the affected hemisphere before and during MCA occlusion. Additionally, in hypertensive rats, chronic infusion with Candesartan for 28 days before MCA occlusion reduced the infarction volume and was associated with an improved cerebral blood flow, especially in the cortex area at the periphery of the infarction [61].

Similarly, chronic treatment with Candesartan improves cerebral vascular self-regulation and diminishes the size of cerebral infarction [62].

Treatment with Candesartan post-stroke in a dose without an effect on arterial blood pressure reduced the volume of infarction and raised the expression of Messenger RNA of the brain-derived neurotrophic factor at 48 h after MCA occlusion [63]. The same aspect was observed in hypertensive rats treated with Candesartan after MCA occlusion, demonstrating the upsurge of brain-derived neurotrophic factor, suggesting a role in neuronal regeneration [64].

Observed in animal models with spontaneous hypertension, media hypertrophy, which appears at the level of large cerebral arteries, can be reversed by long-term blockade of AT1 receptors. Angiotensin receptor blockers can reduce stroke incidence and severity independent of lowering systemic arterial pressure through these mechanisms [20].

The consequence of AT2 receptor stimulation under treatment with ACE inhibitors is related to the stimulation of bradykinin production and secondary vasodilatation by increasing the nitric oxide and the levels of vascular cyclic guanosine-monophosphate (cGMP) [4]. The effect is similar to ACE inhibitors’ action, potentiating the bradykinin effect by inhibiting its degradation.

ACE inhibitors may lead to left ventricular hypertrophy reversibility and the prevention of cardiac remodeling after myocardial infarction.

In a similar pattern, by reducing the hemodynamic load and preserving the atrium’s structural pattern, ACE inhibitors prevent atrial fibrillation, thus reducing the ischemic stroke risk [15,40].

### 3.5. Angiotensin Receptor Antagonists—The Future of RAS-Neuroprotection Axis

C21 is a specific, selective, nonpeptide AT2 receptor antagonist which has properties already proven useful in experimental studies regarding myocardical infarction.

In normotensive rats, systemic and central administration of an AT-2 receptor (compound 21—C21) before and after MCA occlusion reduced the size of the infarction area and improved the neurological deficit [65].

In addition, C21 reduced infarction volume in hypertensive rats when administered centrally for five days before and after MCA occlusion [66].

Another effect of C21 treatment after the administration at 24 h since the oxygen and glucose deprivation event increases the vascular endothelial growth factor (VEGF), promoting angiogenesis. 

Mice with temporary MCA occlusion followed by reperfusion, which received daily treatment for four days with C21, showed improved survival and neurological deficits without impacting the infarction volume. In addition, the number of neurons with apoptosis was significantly lower in the area around the infarction of the mice treated with C21 than in the control group. In the same study, mice with AT-2 receptor knockout showed the administration of C21 did not have any effect, demonstrating the neuroprotective role AT-2 receptors present [67].

In another study, also performed on mice, it was observed that in mice in which they administered C21 immediately after MCA occlusion, a significant reduction of the ischemic area was noted, with improvement of neurological deficit and cerebral blood flow, without any effect on arterial blood pressure. Supplementary C21 treatment reduced the production of pro-inflammatory cytokines TNF-alpha and anionic superoxide [68].

The experimental results provided by using C21 in the embolic stroke rat model showed sensory, motor, and cognitive improvement. Another clinical study used another neuroprotective compound, NA-1, which had similar results when administered to ischemic stroke patients that did not receive thrombolysis.

Apart from the already mentioned advantages of C21 use in ischemic stroke, a reduction of hemorrhagic transformation was also demonstrated on the subjects [69,70].

Furthermore, the combined treatment of C21 and memantine, a drug used in Alzheimer’s disease, showed an increased brain-derived neurotrophic factor value, but without producing any additional effect on the cognitive function [71].

In another experimental study following a translational model of diabetes mellitus to study post-stroke cognitive impairment, after the delayed administration of C21, both a reduction of mortality and an improvement of cognitive deficits was observed. In the same study, the rats underwent a middle cerebral artery occlusion for 1 h, and after three days from the event, they began receiving C21 daily over eight weeks.

Apart from cognitive amelioration, inflammation and demyelination were reduced. Although the number of microglia remained unchanged after C21 treatment, a transition of the activated cells over the M2 phenotype, which has anti-inflammatory properties when compared to the M1 phenotype, was observed. The M1:M2 ratio dropped significantly after receiving treatment with C21 [72].

Another AT2 receptor agonist, CGP42112, reduced the progression of the lesion and improved motor function after the temporary occlusion of MCA. This effect was independent of the change in arterial pressure [73,74].

## 4. Summary of Evidence

A summary of each intervention and effect demonstrated in stroke models can be found in Table 1.

## 5. Conclusions

The control of blood pressure is essential for primary and secondary prevention of ischemic stroke, and it represents an important etiological factor for the reduction of the incidence of hemorrhagic stroke, the current clinical guidelines clearly establishing its role as a major risk factor. One of the benefits of using antihypertensive drugs such as ACE inhibitors or angiotensin II receptor blockers is the reduction of the risk of stroke, independently of their ability to reduce blood pressure.

Although some contradictory data exist, angiotensin II receptor blockers appear to have a superior neuroprotective effect compared to ACE inhibitors, even if the difference is minor. This superior effect does not translate to the incidence rate of stroke, where the two drug classes appear to have similar reduction potential. However, the clinical findings supported by the experimental animal models suggest that these drug classes have other mechanisms of action related to neuroprotection in stroke that is independent of their ability to reduce blood pressure. These properties of ACE inhibitors and angiotensin II receptor blockers seem to be directly linked to the effects of angiotensin II on AT1, AT2, and Mas receptors. Some of these effects consist of neuroregeneration, angiogenesis, increasing the neuronal resistance to hypoxia, reducing the ischemic lesion size, especially in the penumbra, and improving the neurological functions. Nevertheless, all these pathways of action targeting the RAS could create new opportunities for different agonists, such as C21, that could be used to limit the devastating effects of stroke.

## Figures and Tables

**Table 1:** Summary of evidence.

Intervention Performed	Effects Observed	Source
**Angiotensin 2 infusion**	Reduced post-stroke mortality Enhanced cerebral perfusion	Fernandez et al. [55]
**Ang-(1-7) treatment with ischemic stroke**	Decreased microglial activation and neuronal-derived chemokines	Haberl et al. [57]
**Ang-(1-7) treatment with ischemic stroke**	Reduced nitric oxide secretion Decreased level of CXCL12, IL1-beta, IL6, CD11B	Regenhardt et al. [58]
**Ang-(1-7) treatment with ischemic stroke**	Reduction of cerebral infarction volume Improvement of neurological function No effect on blood pressure, heart rate, or cerebral blood flow	Bennion et al. [59]
**Ang-(1-7) treatment with ischemic stroke**	Reduction of cerebral infarction volume Effect was blocked by A779 co-infusion	Meca et al. [34]
**Ang-(1-7) treatment with hemoraghic stroke**	Increased life expectancy Improved neurological function Decreased microglical activation	Regenhardt et al. [60]
**Candesartan treatment before and after MCA occlusion**	Increased cerebral blood flow in affected hemisphere Reduction of cerebral infarction volume	Engelhorn et al. [61]
**Candesartan post-stroke treatment**	No effect on arterial blood pressure Raised expression of mRNA of brain-derived neurotrophic factor	Dai et al. [63]
**Candesartan treatment in hypertensive rats with MCA oclusion**	Raised expression of mRNA of brain-derived neurotrophic factor	Alhusban et al. [64]
**Candesartan chronic treatment**	Improved cerebral vascular self-regulation Reduction of cerebral infarction size	Nishimura et al. [62]
**C21 treatment before and after MCA oclusion**	Reduction of cerebral infarction volume Improved neurological function	Joseph et al. [65]
**C21 treatment 5 days after MCA occlusion**	Reduction of cerebral infarction volume	McCarthy et al. [66]
**C21 treatment after MCA occlusion and reperfusion**	Improved survival rate Improved neurological function Decreased neuron apoptosis rate	Schwengel et al. [67]
**C21 treatment in MCA occlusion**	Reduction of cerebral infarction volume Improved neurological function Reduced production of TNF-alpha and anionic superoxides	Min et al. [68]
**C21 treatment after embolic stroke**	Improved neurological function Reduction of hemorrhagic transformation	Ishrat et al. [69] Hill et al. [70]
**C21 treatment in diabetes mellitus post-stroke model**	Reduced mortality rate Improved cognitive function	Jackson et al. [72]
**CGP42112 treatment in MCA occlusion**	Improved neurological function Reduced lesion progression	McCarthy et al. [73]
